# WALANT Thumb CMC Joint Denervation Under Field Sterility: A Case Report and Description of Technique

**DOI:** 10.1177/22925503261436369

**Published:** 2026-04-09

**Authors:** Alexander Platt, Merry Faye Graff, Brett Ponich, Aaron Knox

**Affiliations:** 1 Cumming School of Medicine, 2129 University of Calgary, Calgary, Alberta, Canada; 2 Division of Plastic and Reconstructive Surgery, 2129 University of Calgary, Calgary, Alberta, Canada; 3 Department of Surgery, 70401 University of Calgary, Calgary, Alberta, Canada

**Keywords:** thumb osteoarthritis, selective denervation, Wide-Awake Local Anesthesia No Tourniquet, hand surgery, thumb carpometacarpal joint, anesthésie locale sur patient éveillé sans usage du garrot, arthrose du pouce, articulation carpométacarpienne du pouce, dénervation sélective, chirurgie de la main

## Abstract

Thumb carpometacarpal (CMC) osteoarthritis is a common condition that can affect a patient's ability to function normally in daily life. Surgical CMC denervation of the thumb has emerged as a less invasive option for patients who want to recover and return to activity sooner. Here, we report the use of the Wide-Awake Local Anesthesia No Tourniquet technique for first CMC denervation under field sterility.

## Résumé

L'arthrose de l'articulation carpométacarpienne (CMC) est une affection courante qui nuit au fonctionnement quotidien normal. La dénervation chirurgicale de l'articulation CMC du pouce se révèle une option moins invasive pour les personnes qui souhaitent se rétablir et reprendre leurs activités plus rapidement. Les auteurs rendent compte de l'utilisation de la technique de WALANT (*Wide Awake Local Anesthesia No Tourniquet*, ou anesthésie locale sur patient éveillé sans usage du garrot) pour dénerver la première articulation CMC sous champ stérile.

## Background

The thumb is essential for hand function, enabling grip, pinch, and fine motor movement. Osteoarthritis (OA) of the thumb carpometacarpal (CMC) joint is a common site of hand OA, affecting 15% of women and 7% of men.^1^ First CMC OA may present with debilitating pain, decreased grip strength, and reduced mobility that limit daily activities.^2^ Nonoperative treatments such as anti-inflammatories, physical therapy, splinting, or steroid injections may provide relief.^2^ Many surgical options (arthrodesis, arthroscopy, implant arthroplasty, trapeziectomy, etc) have been described with high success rates.^3^ Complication rates vary by procedure, and these surgeries typically require prolonged immobilization and rehabilitation. An alternative, less invasive surgical option is selective denervation of the first CMC joint, targeting sensory nerve branches to the joint. This method effectively improves symptomatic arthritis while reducing rehabilitation and immobilization.^4–7^

Wide-Awake Local Anesthesia No Tourniquet (WALANT) surgery has gained acceptance as a safe and effective modality to treat varying hand pathologies.^8^ We have previously described the use of this technique for proximal row carpectomy and scaphoid fixation.^9,10^ Here, we describe selective denervation of the thumb CMC joint under WALANT with field sterility for the management of OA.

## Methods

Electronic medical records were used to access imaging and pertinent medical history. The patient provided verbal informed consent to publish her images and information.

### Initial Consult

We report the case of an 81-year-old, right-hand-dominant female who presented to hand clinic with swelling and pain at the base of her right thumb, first noted in 2019. She previously worked as an accountant. Her past medical history was significant for a thyroidectomy, well-managed on levothyroxine. She was otherwise healthy, with no history of smoking or diabetes and denied any prior hand injuries.

**Figure 1.**
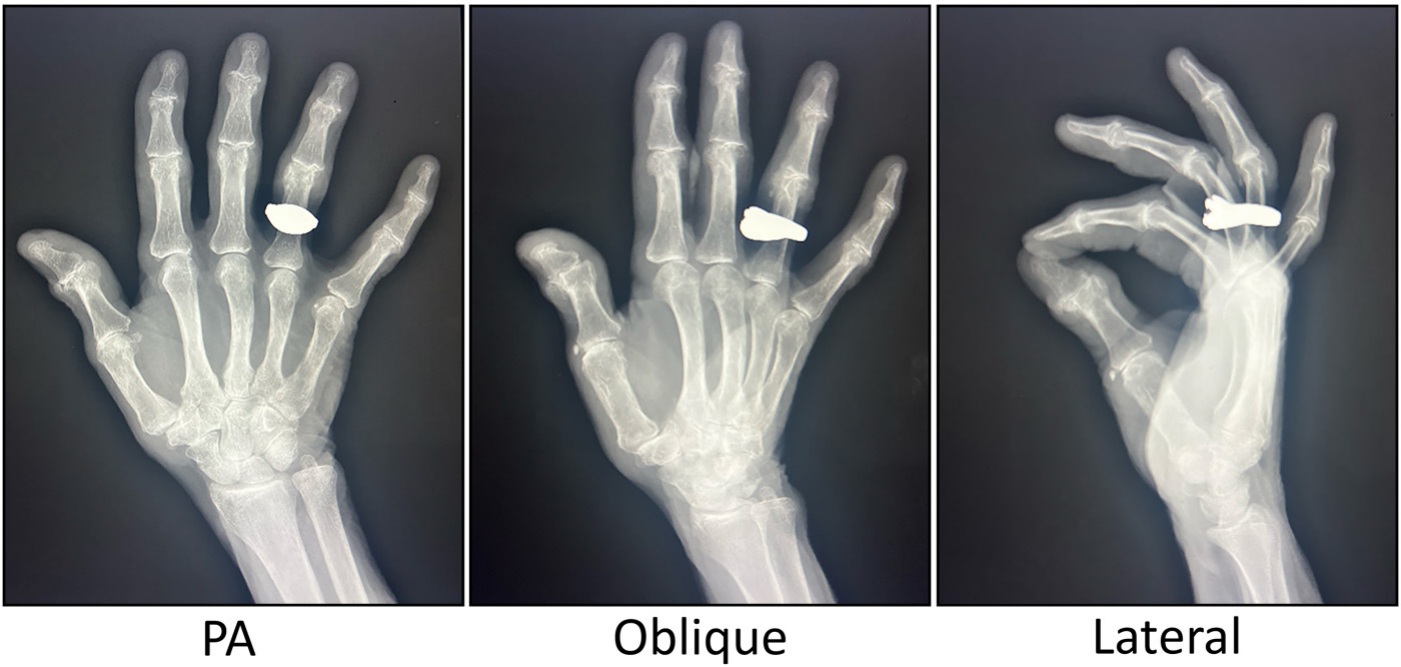
Radiographs of the right hand from September 2023 demonstrating moderate osteoarthritis in the right thumb CMC joint. CMC, carpometacarpal; PA, posteroanterior.

The patient described daily pain and difficulty performing tasks such as opening jars or holding objects. She reported dropping items and bouts of shooting pain. Previous treatments included 6 corticosteroid injections with diminishing relief. Hand radiographs demonstrated moderate joint space narrowing and subchondral sclerosis consistent with Eaton Stage^11^ 2 of the right CMC joint (Figure 1). On examination, there was a shoulder step-off sign, a positive grind test, and the patient was tender to palpation, with swelling and crepitus at the base of the thumb.

Treatment options were reviewed, including the risks and benefits of a trapeziectomy and CMC denervation. Ultimately, the patient chose surgical denervation of the first CMC joint using the WALANT technique for its quicker recovery, reduced downtime, and the benefit of performing it with local anesthesia rather than general anesthesia. The patient was informed that trapeziectomy remains an option if denervation fails.

### Procedure Set-Up

The patient was placed on the operating table with their right hand extended on an arm board. Approximately 20cc of 1% lidocaine with epinephrine was used for a field block along with the dorsal and volar aspects of the thumb. Additional freezing was used for a wrist block. Equipment for the procedure was prepared while waiting for the local anesthetic to take effect (∼15 min).

**Figure 2.**
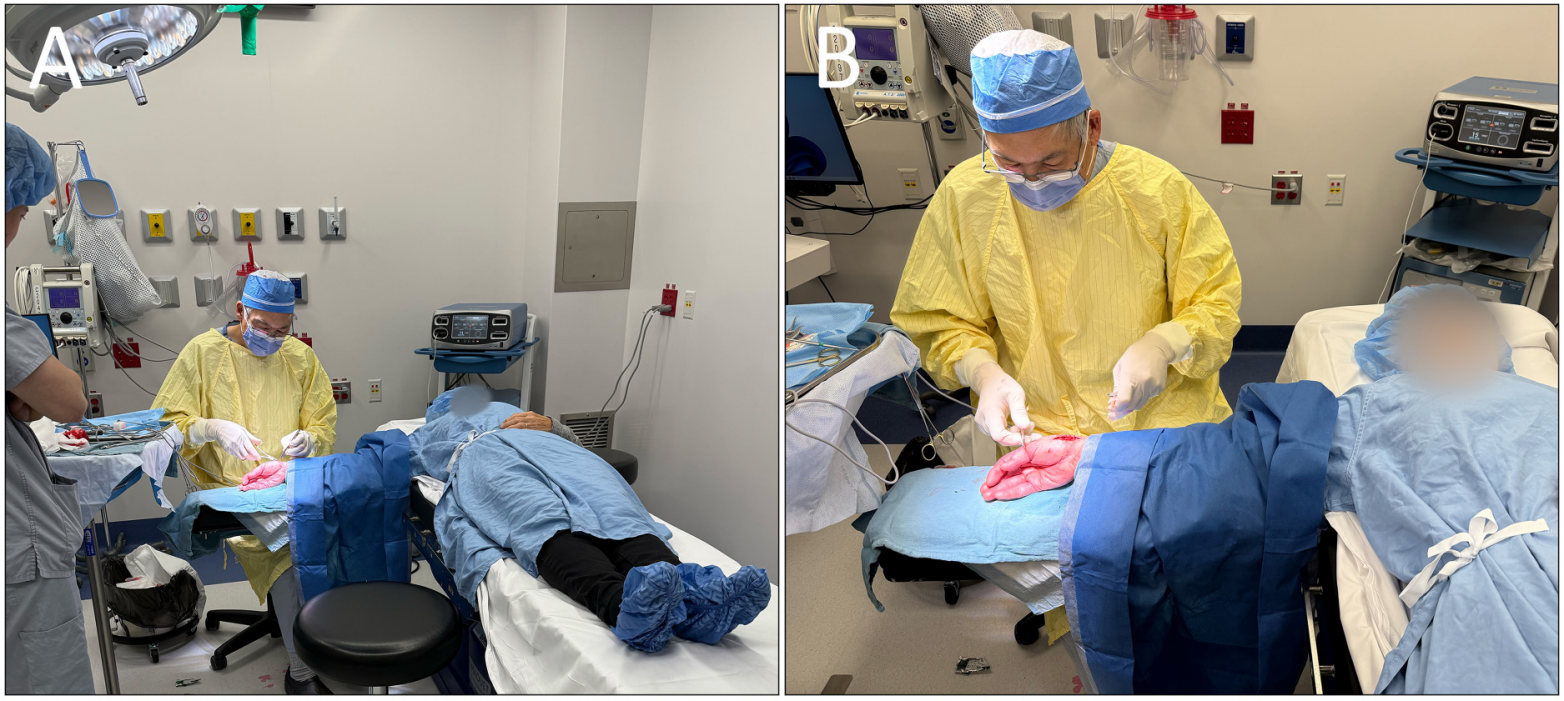
(A) Minor surgery suite set up and (B) patient position for the procedure. Please note that the operating physician is wearing a gown for warmth; this is not required for Wide-Awake Local Anesthesia No Tourniquet procedures.

The hand was sterilized from the proximal forearm to the fingertips using chlorhexidine and draped using 2 sterile towels and a single disposable sterile drape (Figure 2). Note that the operating surgeon does not require sterile surgical attire beyond sterile gloves. The patient's numbness was confirmed using a grind test and by carefully probing the incision area, with additional PRN local anesthetic given as needed before and during the procedure.

### Operative Technique

**Figure 3.**
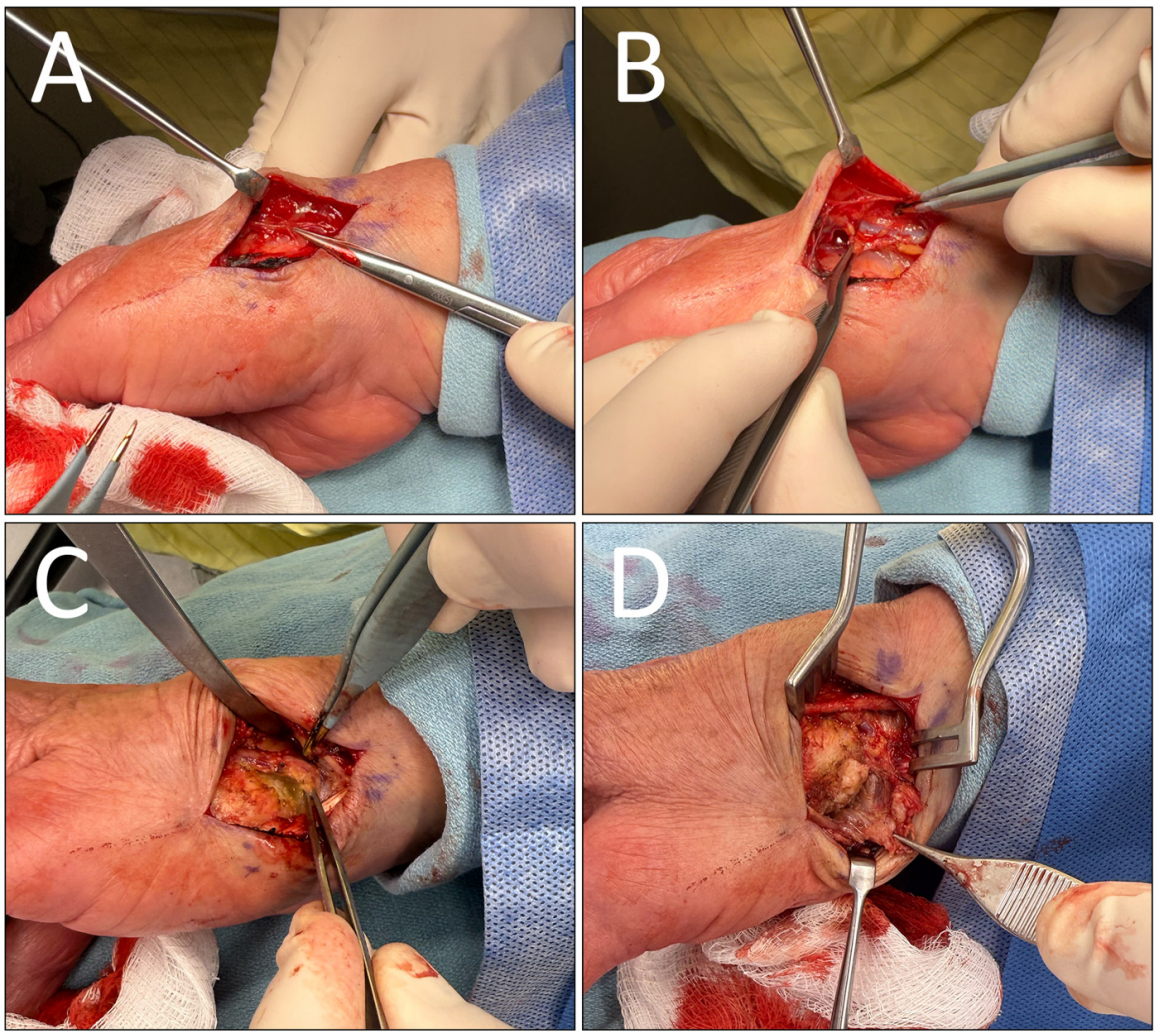
Intraoperative photos of denervation showing (A) articular branches of the dorsal radial sensory nerve (B) visualized cephalic vein, (C) thumb CMC joint capsule, (D) thenar musculature dissected from the base of the first metacarpal. CMC, carpometacarpal.

A straight 3 cm incision was made along with the volar radial side of the thumb at the metacarpal base and CMC joint. We then bluntly dissected down to the extensor tendons. As the flap was lifted off the extensor fascia toward the base of the second metacarpal, the cutaneous nerves of the skin flap were seen extending toward the capsule within the areolar tissue. These nerves were identified as the articular branches of the dorsal radial sensory nerve and divided (Figure 3A).

The area between the abductor pollicis longus (APL) and extensor pollicis brevis tendons was exposed and dissected to access the floor of the snuff box. As the radial artery was dissected off the dorsal capsule of the CMC joint and the scaphotrapeziotrapezoidal joint, the articular branches from the lateral antebrachial cutaneous nerve were divided. The cephalic vein was identified (Figure 3C), and the radial artery was carefully mobilized and protected. The volar aspect of the dorsal capsule floor was then cauterized with bipolar (Figure 3C).

Next, we began dividing an additional articular branch of the dorsal radial sensory nerve by elevating a volar flap. The thenar musculature was lifted from the APL tendon insertion, approaching the flexor carpi radialis tendon sheath and volar beak ligament. The volar capsule was thoroughly cauterized to ensure any articular branches from the thenar and palmar cutaneous branches of the median nerve were divided (Figure 3D). The thenar musculature was repaired with 3-0 Vicryl. Layered skin closure was performed with 4-0 Monocryl, and a nonadherent dressing was applied.

Overall, the operation lasted approximately 30 minutes and was well tolerated by the patient, with no pain reported during the procedure. The patient was seen 2 weeks postoperatively with no concerns.

## Discussion

WALANT surgery under field sterility has gained popularity for treating hand pathologies. This technique reduces the time and materials needed in a main operating theatre, thereby reducing environmental impact and overall cost. Unlike previously described awake denervation techniques,^12^ this procedure was performed without a tourniquet, eliminating tourniquet-related discomfort and ischemic pain, which are limiting factors in awake hand surgery.^13^ Using local anesthesia allows for intraoperative communication with patients and makes it easier to share postoperative care instructions and follow-up steps, saving time. For patients suffering from first CMC OA, it presents a less invasive alternative that can be performed in a minor surgery setting without the need for general anesthesia or extensive operating room time. This is particularly beneficial for individuals with comorbid conditions that may increase the risk of general anesthesia. The shorter recovery time and absence of an immobilization period allow patients to return to work or normal activities sooner than with other operations. Additionally, this technique preserves the integrity of the joint, ensuring patients remain eligible for further treatment.
